# Electrical Remodelling in Cardiac Disease

**DOI:** 10.3390/cells12020230

**Published:** 2023-01-05

**Authors:** Ursula Ravens, Rémi Peyronnet

**Affiliations:** Institute for Experimental Cardiovascular Medicine, University Heart Center Freiburg-Bad Krozingen and Faculty of Medicine, University of Freiburg, 79110 Freiburg im Breisgau, Germany

## 1. Introduction

The human heart responds to various diseases with structural, mechanical, and electrical remodelling processes. These changes may initially compensate functional impairment, however, in the long run they may promote disease progression and aggravate functional decline. The various remodelling processes are highly interrelated. Nevertheless, electrical remodelling appears to be the most dangerous one in the heart because of its propensity to deteriorate into life-threatening arrhythmias. The mechanisms by which electrical remodelling may lead to malignant arrhythmias are not fully understood. In order to provide better risk prediction for arrhythmic events in cardiac disease, we need to advance our understanding of electrical remodelling at the molecular, cellular, and whole-organ level in the context of different cardiac pathologies. The purpose of the collection of articles in this Special Issue of Cells is to provide an overview of molecular, micro- and macroscopic events during electrical remodelling in the context of different heart diseases. The long-term aspiration is to create a framework for the development of better protection against lethal arrhythmias.

## 2. Cardiac Electrical Activity and Disease-Induced Electrical Remodelling

Arrhythmias are triggered by focal events (early or delayed after-depolarizations) occurring in a susceptible substrate of non-synchronous action potential conduction (e.g., focal [scar] or diffuse fibrosis, weak cellular coupling) [1]. During an action potential the cardiomyocyte is depolarized from a negative resting membrane potential by an inward Na^+^ current, the plateau phase is maintained by the slowly inactivating L-type Ca^2+^ current (I_Ca,L_), and repolarization back to the resting potential is brought about by various K^+^ currents. The net cation movements that take place during such an action potential are compensated by the Na^+^/K^+^-ATPase and Na^+^/Ca^2+^ exchanger, both of which are electrogenic and generate a membrane current of their own. Increases or decreases in any of these current densities alter the shapes of the action potentials that may lead to arrhythmias.

Moreover, disease-associated perturbations of the cellular Ca^2+^ homeostasis can have profound arrhythmogenic effects. During systole Ca^2+^ influx via L-type calcium channels (I_Ca,L_) triggers further release of Ca^2+^ from the sarcoplasmic reticulum via type 2 ryanodine receptors (RyR2) for contractile activation. The cardiomyocytes relax when the high systolic Ca^2+^ concentration is lowered by Ca^2+^ reuptake into the sarcoplasmic reticulum, and by extrusion from the cells via the Na^+^/Ca^2+^ exchanger [2]. Spontaneous release of Ca^2+^ from the SR [3] or enhanced dissociation of Ca^2+^ from cardiac myofilaments [4] are arrhythmogenic because they may cause excessive activation of the Na^+^/Ca^2+^ exchanger during diastole [5]. The resulting depolarization may reach threshold of a propagated action potential, thus giving rise to extrasystoles. 

Prominent examples of disease-induced electrical remodelling are prolongation of action potentials in heart failure [6], or shortening of the atrial refractory period and loss of rate-dependent adaptation in atrial fibrillation [7]. The literature abounds with reports on functional changes or altered expression levels in the canonical cation channels. In addition, mutations in the genes encoding for ion channels can cause either loss or gain of function, and both changes have been associated with arrhythmias. 

## 3. Ion Channels

The advent of an improved patch-clamp technique for high-resolution current recording from single cells [8] has enabled detailed studies of the dynamics of membrane currents underlying the cardiac action potential. This technique requires dissociation of cardiac tissues into single cardiomyocytes, that is not always achieved with satisfactory yields, especially when human samples are involved. The first article in the current Special Issue of *Cells* describes a method of freshly isolating primary atrial and ventricular cardio-myocytes based on tissue slices [9]. Structural and functional properties of the cardiomyocytes are well preserved when dissociated over three consecutive days after obtaining the parent tissue, thus allowing for repeated measurements and collection of larger data sets from the same heart.

Physiological channel function is tightly linked to their proper incorporation into the sarcolemma. Defects in intracellular trafficking and sarcolemmal organization of ion channels are associated with electrical remodelling in cardiac disease [10]. The authors explain the prominent role of the Rab family of small GTPases in coordinating the many steps of cytosolic ion channel trafficking, recycling and degeneration, and how molecular motors such as kinesin and dynein are required for moving ion channel-containing vesicles along cellular microtubules. In the heart, numerous members of the protein family of membrane-associated guanylate kinase (MAGUK) are expressed and form macromolecular complexes with for instance Na^+^ channels, directing them to distinct sarcolemmal locations such as T-tubules, lateral membrane or intercalated discs. Since Na^+^ channels play a dominant role in conduction, any default in this trafficking system can result in profound arrhythmias. The authors continue with a detailed overview of the trafficking machinery for various K^+^ channels in which the balance between antegrade and retrograde trafficking is altered during atrial fibrillation-triggered remodelling. 

While there is a rich knowledge on voltage-dependent Na^+^, Ca^2+^ and K^+^ channels, much less is known about the large family of two pore K^+^ (K_2P_) channels that conduct ‘leak’ or ‘background’ current. Most of these channels exhibit little voltage dependence and are activated by other physical factors such as temperature, stretch, pH, lipids or drugs. In their review Wiedmann et al. contrast expression of K_2P_ channels in human right atrial and left ventricular tissue and provide a valuable compilation of the nomenclature and crystal structure of the 15 members of this channel family [11]. In addition, they provide a comprehensive tabular overview of published evidence for cardiac expression of K_2P_ channel subunits at mRNA or protein level in different species, followed by a compilation of drugs previously reported to inhibit or activate these channels. With respect to electrical remo-delling in cardiac disease, expression of K_2P_3.1 (TASK-1) is of special interest: besides being more abundant in atria than ventricles [12], this channel is up-regulated in atrial fibrillation (contributing to the shortened action potential) and down-regulated in heart failure where action potentials are prolonged [13]. Moreover, in a pig model of atrial fibrillation, pharmacological inhibition of TASK-1 reverses atrial action potential shortening [11].

Other ‘non-canonical’ ion channels involved in cardiac electrophysiology belong to the transient receptor potential (TRP) family, of which the melastatin-related subfamily member 4 (TRPM4) is functionally expressed in the heart, especially in the atria. This Ca^2+^-activated nonselective cation channels is permeable to the monovalent ions K^+^ and Na^+^ but impermeable to Ca^2+^, and is suggested to be involved in cardiac arrhythmogenesis [14]. TRPM4 channels are regulated by the intracellular Ca^2+^ concentration and contribute to depolarizing currents at negative and hyperpolarizing currents at positive membrane potentials. Hyperaldosteronemia was associated with atrial fibrillation in rats [15]. The application of this model to control and TRPM4 knock-out mice suggests a possible arrhythmogenic role of TRPM4. Indeed TRPM4 deficient mice showed a decreased aldosteron-induced early and delayed afterdepolarizations, and hence lowered the propensity to develop atrial arrhythmias [16]. 

## 4. Calcium-Handling Proteins

In atrial fibrillation, phosphorylation of Ca^2+^ handling proteins is altered at multiple sites [17]. One explanation for these findings is provided by the demonstration that cytosolic cAMP levels are differentially regulated in the various Ca^2+^-handling compartments in sinus rhythm and AF, contributing to AF-induced electrical remodelling [18]. The local c-AMP concentration is determined by the balance between c-AMP formation by activation of adenylyl cyclase via G-protein-coupled receptors, mainly β_1_- and β_2_-adrenoceptors, and c-AMP breakdown by phosphodiesterases, suggesting that these proteins are also subject to compartmentation. β-Adrenoceptors and ion channels are located in close vicinity to each other within the transverse-axial tubule system. Therefore, de-tubulation of cardiomyocytes associated with cardiac disease is linked to electrical remodelling with arrhythmogenic potential [19]. Disruption of the transverse-axial system as observed for instance in heart failure or atrial fibrillation is associated with dysfunction of Ca^2+^ handling proteins that may lead to Ca^2+^-driven proarrhythmic effects [20]. 

## 5. Conduction

Conduction velocity has a major impact on re-entry. Han et al. report experimental approaches for the measurement of conduction velocity in myocardial tissue or whole hearts and emphasize that correct measurement is a prerequisite for estimating its arrhythmogenic role. Since experimental methods have various limitations, computational models can be used for illustrating the effects of laminar discontinuities and cell strands on conduction velocity distributions [21].

Proarrhythmic electrical remodeling encompasses also connexins, the proteins forming gap junction channels for cell-cell coupling. Clustering of gap junctions at the cell ends ensures fast anisotropic conduction in the longitudinal direction of myocardial cells. In atrial fibrillation or heart failure these channels are downregulated and redistributed from the intercalated discs to the lateral cell membrane, resulting in transverse conduction and reduced conduction velocity [22]. Desynchronisation of electrical activity promotes and sustains arrhythmias. Gap junctions are also discussed as pharmacological targets to treat arrhythmias [22]. 

## 6. Fibrosis

In addition, electrical connections between myocytes can be disrupted by various distinctly different types of fibrous tissue that develop in the aged and diseased myocardium. The relation of fibrosis and substrate for arrhythmias is reviewed by Verheule and Schotten [23]. Accordingly, fibrosis promotes both major contributors to arrhythmias, ectopy and re-entry: Low, heterogenous coupling of a cluster of injured cells with the surrounding healthy cardiomyocytes is viewed to increase the likelihood of initiating an ectopic propagated action potential, and re-entry is facilitated by diminished conduction velocity.

## 7. Arrhythmias Associated with Specific Cardiac Diseases

In dogs, rapid atrial pacing over several weeks produces a substrate of high susceptibility to atrial fibrillation induced by programmed stimulation [24]. This model does not only mimic electrical remodelling but also reveals severe structural, architectural and contractile changes affecting the atria, later defined as ‘atrial cardiomyopathy’ [25]. Marked swelling of mitochondria and their structural disruption are interpreted as signs of “oxidative stress”. Though not specific to AF, this central pathophysiological mechanism is discussed as the main contributor to development and progression of AF, and its link to multiple signal transduction cascades including inflammatory pathways that are disrupted in AF is unravelled [26].

An in-depth summary of genetic and acquired pathophysiological causes of sick sinus syndrome, Brugada syndrome, and atrial fibrillation reviews preclinical animal and cell models as well as computational simulations for these arrhythmias [27]. In chronic heart failure, both ventricular tachyarrhythmia and atrial fibrillation are common arrhythmias. Despite opposite remodelled action potential phenotypes in heart failure and atrial fibrillation (i.e., prolongation vs. shortening of action potential duration), the tachyarrhythmias in ventricles and atria share the common basic proarrhythmic mechanisms, namely ectopic activity and re-entry promoted by fibrosis. These issues are expertly explained for failing hearts in different experimental models by Husti et al. [28]. Dilated cardiomyopathy is a structural heart disease based on genetic, acquired or mixed cause. The basic proarrhythmic pathomechanism also includes prolonged ventricular action potential due to remodelling of ion channels and myocardial fibrosis. Mutations in a large number of genes that encode not only for ion channels and intercellular junction molecules but also for contractile and cytoskeleton proteins have been identified in patients with dilated cardiomyopathy. In addition, electrical remodelling is affected by chromosomal alterations without changes in DNA sequence, referred to as epigenetic changes [29]. Hypertrophic cardiomyopathy is also burdened with life-threatening ventricular arrhythmias. Electrical remodelling includes increased late Na^+^ current and I_Ca,L_ thereby enhancing early and delayed afterdepolarizations. Mutations in the gene encoding β-cardiac myosin heavy chain increase myofilament Ca^2+^ sensitivity which leads to dysfunctional diastolic Ca^2+^ handling and further increases arrhythmogenic risk [30]. Amoni and Sipido approach their review of ventricular arrhythmias in ischemic cardiomyopathy from a therapeutic viewpoint [31]. They provide novel mechanistic insight to molecular alterations of the vulnerable myocardial infarction boarder zone with the implicit aim to arrive at new avenues for treatment.

## 8. Computational Simulation of Electrical Remodelling

Computational models help to understand complex pathophysiological mechanisms that are not accessible to biological experimentation because they allow control of a wide range of parameters [32]. Electrical propagation within the heart is additionally affected by direct coupling of fibroblasts with cardiomyocytes [33]. How this electrical coupling between non-myocytes and myocytes might impact atrial fibrillation is examined in a purely in-silico approach [34]. In this computational model, addition of an I_Ca,L_ to the electrophysiology of the non-myocytes can trigger automaticity propagated in the surrounding atrial tissue.

In order to prevent rather than treat life-threatening ventricular arrhythmias in cardiac disease, risk prediction of adverse events is sorely needed. Due to the multifactorial disease mechanisms also involving genetic factors, exuberant amounts of data accumulate that require machine learning techniques for meaningful conclusions. Here, Yang et al. employ a combination of artificial intelligence-assisted identifications of single nucleotide polymorphisms and clinical parameters to identify asymptomatic high-risk subjects that are predisposed to heart failure [35].

## 9. Outlook

This Special Issue of *Cells* on ‘*Electrical Remodelling in Cardiac Disease*’ cannot (and did not aim to) provide a comprehensive description of electrical remodelling. Many topics are left for future consideration, such as crosstalk between different cardiac cell populations via extracellular vesicles [36] or the impact of exercise [37] and ageing [38] on cardio-vascular remodelling processes. In addition, amazing advances are expected through computer simulations and artificial intelligence while they become increasingly sophisticated.

## References

[1] Tse G. (2016). Mechanisms of cardiac arrhythmias. J. Arrhythmia.

[2] Bers D.M. (2002). Sarcoplasmic reticulum Ca release in intact ventricular myocytes. Front. Biosci. A J. Virtual Libr..

[3] Berlin J.R., Cannell M.B., Lederer W.J. (1989). Cellular origins of the transient inward current in cardiac myocytes. Role of fluctuations and waves of elevated intracellular calcium. Circ. Res..

[4] Ter Keurs H.E., Wakayama Y., Miura M., Shinozaki T., Stuyvers B.D., Boyden P.A., Landesberg A. (2006). Arrhythmogenic Ca^2+^ release from cardiac myofilaments. Prog. Biophys. Mol. Biol..

[5] Fujiwara K., Tanaka H., Mani H., Nakagami T., Takamatsu T. (2008). Burst emergence of intracellular Ca2+ waves evokes arrhythmogenic oscillatory depolarization via the Na^+^-Ca^2+^ exchanger: Simultaneous confocal recording of membrane potential and intracellular Ca^2+^ in the heart. Circ. Res..

[6] Beuckelmann D.J., Näbauer M., Erdmann E. (1993). Alterations of K+ currents in isolated human ventricular myocytes from patients with terminal heart failure. Circ. Res..

[7] Wijffels M.C., Kirchhof C.J., Dorland R., Allessie M.A. (1995). Atrial fibrillation begets atrial fibrillation. A study in awake chronically instrumented goats. Circulation.

[8] Hamill O.P., Marty A., Neher E., Sakmann B., Sigworth F.J. (1981). Improved patch-clamp techniques for high-resolution current recording from cells and cell-free membrane patches. Pflug. Arch. Eur. J. Physiol..

[9] Greiner J., Schiatti T., Kaltenbacher W., Dente M., Semenjakin A., Kok T., Fiegle D.J., Seidel T., Ravens U., Kohl P. (2022). Consecutive-day ventricular and atrial cardiomyocyte isolations from the same heart: Shifting the cost-benefit balance of cardiac primary cell research. Cells.

[10] Blandin C.E., Gravez B.J., Hatem S.N., Balse E. (2021). Remodeling of ion channel trafficking and cardiac arrhythmias. Cells.

[11] Wiedmann F., Frey N., Schmidt C. (2021). Two-pore-domain potassium (k_2p−_) channels: Cardiac expression patterns and disease-specific remodelling processes. Cells.

[12] Schmidt C., Wiedmann F., Voigt N., Zhou X.B., Heijman J., Lang S., Albert V., Kallenberger S., Ruhparwar A., Szabo G. (2015). Upregulation of K_2P_3.1 K+ current causes action potential shortening in patients with chronic atrial fibrillation. Circulation.

[13] Schmidt C., Wiedmann F., Zhou X.B., Heijman J., Voigt N., Ratte A., Lang S., Kallenberger S.M., Campana C., Weymann A. (2017). Inverse remodelling of K2P3.1 K+ channel expression and action potential duration in left ventricular dysfunction and atrial fibrillation: Implications for patient-specific antiarrhythmic drug therapy. Eur. Heart J..

[14] Guinamard R., Demion M., Magaud C., Potreau D., Bois P. (2006). Functional expression of the TRPM4 cationic current in ventricular cardiomyocytes from spontaneously hypertensive rats. Hypertension.

[15] Reil J.C., Hohl M., Selejan S., Lipp P., Drautz F., Kazakow A., Münz B.M., Müller P., Steendijk P., Reil G.H. (2012). Aldosterone promotes atrial fibrillation. Eur. Heart J..

[16] Simard C., Ferchaud V., Sallé L., Milliez P., Manrique A., Alexandre J., Guinamard R. (2021). TRPM4 participates in aldosterone-salt-induced electrical atrial remodeling in mice. Cells.

[17] El-Armouche A., Boknik P., Eschenhagen T., Carrier L., Knaut M., Ravens U., Dobrev D. (2006). Molecular determinants of altered Ca^2+^ handling in human chronic atrial fibrillation. Circulation.

[18] Reinhardt F., Beneke K., Pavlidou N.G., Conradi L., Reichenspurner H., Hove-Madsen L., Molina C.E. (2021). Abnormal calcium handling in atrial fibrillation is linked to changes in cyclic amp dependent signaling. Cells.

[19] Wright P.T., Gorelik J., Harding S.E. (2021). Electrophysiological Remodeling: Cardiac T-tubules and ß-adrenoceptors. Cells.

[20] Zhang X., Smith C.E.R., Morotti S., Edwards A.G., Sato D., Louch W.E., Ni H., Grandi E. (2022). Mechanisms of spontaneous Ca^2+^ release-mediated arrhythmia in a novel 3D human atrial myocyte model: II Ca^2+^ -handling protein variation. J. Physiol..

[21] Han B., Trew M.L., Zgierski-Johnston C.M. (2021). Cardiac conduction velocity, remodeling and arrhythmogenesis. Cells.

[22] Dhein S., Salameh A. (2021). Remodeling of cardiac gap junctional cell-cell coupling. Cells.

[23] Verheule S., Schotten U. (2021). Electrophysiological consequences of cardiac fibrosis. Cells.

[24] Morillo C.A., Klein G.J., Jones D.L., Guiraudon C.M. (1995). Chronic rapid atrial pacing. Structural, functional, and electrophysiological characteristics of a new model of sustained atrial fibrillation. Circulation.

[25] Goette A., Kalman J.M., Aguinaga L., Akar J., Cabrera J.A., Chen S.A., Chugh S.S., Corradi D., D’Avila A., Dobrev D. (2016). EHRA/HRS/APHRS/SOLAECE expert consensus on atrial cardiomyopathies: Definition, characterisation, and clinical implication. J. Arrhythmia.

[26] Goette A., Lendeckel U. (2021). Atrial cardiomyopathy: Pathophysiology and clinical consequences. Cells.

[27] Iop L., Iliceto S., Civieri G., Tona F. (2021). Inherited and acquired rhythm disturbances in sick sinus syndrome, brugada syndrome, and atrial fibrillation: Lessons from preclinical modeling. Cells.

[28] Husti Z., Varró A., Baczkó I. (2021). Arrhythmogenic remodeling in the failing heart. Cells.

[29] Mages C., Gampp H., Syren P., Rahm A.K., André F., Frey N., Lugenbiel P., Thomas D. (2021). Electrical ventricular remodeling in dilated cardiomyopathy. Cells.

[30] Santini L., Coppini R., Cerbai E. (2021). Ion channel impairment and myofilament Ca^2+^ sensitization: Two parallel mechanisms underlying arrhythmogenesis in hypertrophic cardiomyopathy. Cells.

[31] Amoni M., Dries E., Ingelaere S., Vermoortele D., Roderick H.L., Claus P., Willems R., Sipido K.R. (2021). Ventricular arrhythmias in ischemic cardiomyopathy-new avenues for mechanism-guided treatment. Cells.

[32] Heijman J., Sutanto H., Crijns H., Nattel S., Trayanova N.A. (2021). Computational models of atrial fibrillation: Achievements, challenges and perspectives for improving clinical care. Cardiovasc. Res..

[33] Quinn T.A., Camelliti P., Rog-Zielinska E.A., Siedlecka U., Poggioli T., O’Toole E.T., Knopfel T., Kohl P. (2016). Electrotonic coupling of excitable and nonexcitable cells in the heart revealed by optogenetics. Proc. Natl. Acad. Sci. USA.

[34] Sánchez J., Trenor B., Saiz J., Dössel O., Loewe A. (2021). Fibrotic remodeling during persistent atrial fibrillation: In silico investigation of the role of calcium for human atrial myofibroblast electrophysiology. Cells.

[35] Yang N.I., Yeh C.H., Tsai T.H., Chou Y.J., Hsu P.W., Li C.H., Chan Y.H., Kuo L.T., Mao C.T., Shyu Y.C. (2021). Artificial intelligence-assisted identification of genetic factors predisposing high-risk individuals to asymptomatic heart failure. Cells.

[36] Martins-Marques T., Girão H. (2022). The good, the bad and the ugly: The impact of extracellular vesicles on the cardiovascular system. J. Physiol..

[37] Perkins D.R., Talbot J.S., Lord R.N., Dawkins T.G., Baggish A.L., Zaidi A., Uzun O., Mackintosh K.A., McNarry M.A., Cooper S.M. (2022). The influence of maturation on exercise-induced cardiac remodelling and haematological adaptation. J. Physiol..

[38] Harraz O.F., Jensen L.J. (2021). Vascular calcium signalling and ageing. J. Physiol..

